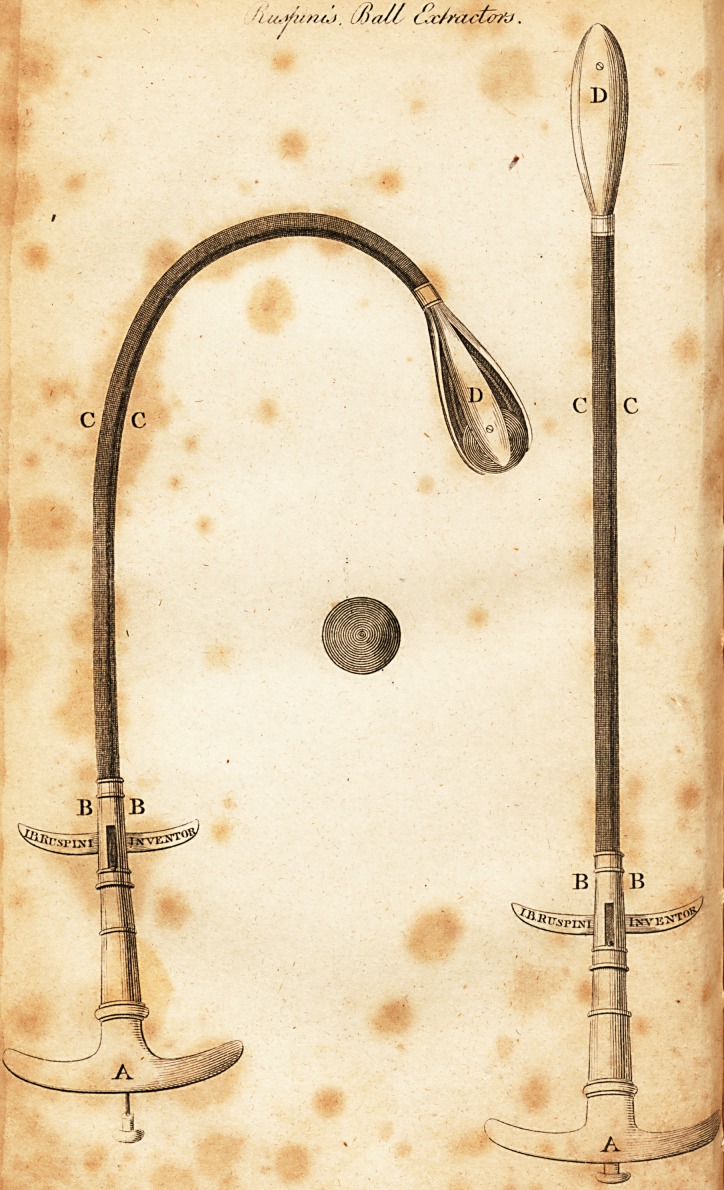# A Brief Description of a Newly-Invented Instrument, for the Extraction of Balls from Gun-Shot Wounds

**Published:** 1806-05

**Authors:** J. B. Ruspini

**Affiliations:** Surgeon-Dentist to His Royal Highness the Prince of Wales


### *A Brief Description of a newly-invented Instrument, for the Extraction of Balls from Gun-Shot Wounds. By* J. B. RUSPINI, *Surgeon-Dentist to His Royal Highness the Prince of Wales.*

( With Engravings. )

THE enlargement of Gun-shot Wounds, for the purpose of extracting the ball, has been acknowledged by many eminent surgeons to be an evil which, from the nature of the probe and forceps at present in use, it has been hitherto impossible to prevent. Nothing can be more true, as a general proposition, than that the larger the wound, the more difficult it is to cure; whilst by the increased loss of blood, which must unavoidably follow a dilatation of the original wound, the patient is frequently much weakened, and rendered less able to contend with the effects of the injury which he has unhappily sustained. It is not, therefore, an object of small importance, if the means can be devised devised by which the offending matter may be extracted without the wound being rendered greater in the endeavour to cure it. The probe and the forceps hitherto in use have been found, in many instances, injurious in their operation, as they frequently still further lacerate the part, and often bring away some of the integuments along with the ball. This injury to the patient has been still more seriously felt when the ball has taken an oblique direction. It is not necessary here to enter into any detail of the nature of Gun-shot Wounds, as they have already been much more ably treated upon by others; my only object is to induce those gentlemen who practice as surgeons, to make trial of an instrument, which, from the experiments that have already been made of its utility, appears, I think, calculated to assist materially in the cure of that description of wounds to which I have alluded.

The peculiar properties of the instrument which I have ventured to bring forward for public investigation are, that it acts as a probe as well as forceps, and does not, when used, dilate the wound or lacerate the parts. In the first place, when the ball takes a straight direction, this instrument, on being introduced, discovers the depth of the wound, and immediately on coming into contact with the ball, the secret springs act by opening the claws, which immediately close firmly on the matter to be extracted; by which means it is easily brought out, whilst the parts originally wounded receive no further injury from its action. In the last case, where a ball takes a curved direction, I have prepared an instrument of a similar nature, but flexible, which follows the ball, and, seizing it in like manner, brings it out likewise without further injury to the parts wounded.

Many eminent surgeons have signified their approbation of the instrument, and have declared its superiority to the forceps now in use; and I am convinced the best explanation of the utility of the instrument will be furnished by experience.

The use of the instrument is not, however, confined to Gun-shot Wounds; it will be found of equal utility in Lithotomy and Bronchotomy. I have every reason to believe that it will be found of the greatest use in extracting a stone from the bladder, or any hard substance from the throat; as in either case it will act precisely in the same manner before described, without lacerating the parts affected. In any case, in short, where it is requisite to extract a hard substance, the instrument may be used with a similar effect.

It

It is of no small moment, therefore, to the public, that a fair trial should be made of the instrument, as in the event of its successful application, it may be productive in many cases of incalculable good. In speaking thus, far be it from me to make any peculiar claim to merit on account of the discovery: my only request is, that it may be tried in the fair scale of experiment; in which case, I flatter myself it will frequently be found of considerable utility.

I have purposely avoided entering into a minute description of Gun-shot Wounds, or of those cases in which I conceive this instrument might be found useful, as that would lead me into far too wide a field, and would be in a great measure foreign to the subject of the present pamphlet. I only think it necessary to press upon the attention of my readers, that it is of the greatest importance that all wounds and all diseases, requiring surgical operation, should be cured with as little pain as possible to the patient, and without any unnecessary laceration of the parts, already too deeply affected. It is in this point of view I have ventured to recommend the Instrument which I have invented for general use; and without entering into any detailed comparison of its properties with those of other instruments now employed, I leave it to speak for itself.

In the annexed Plate is a delineation of two Instruments, of which, fig. 1 is to be used when a ball takes a straight direction, or when the substance to be extracted lies in a similar line. A is the handle; BB the secret springs, by means of which the claws open when they come in contact with the ball; CC the straight probe; and D the claws, which close immediately when they have seized the ball or other substance.

Fig. 2, is an Instrument to be employed when the ball has taken a curved direction. A and BB as before; CC a flexible probe; and D the claws, which operate in the same manner as above mentioned, by immediately closing when the ball is within them.

The method of using the Instrument is simply as follows. It is introduced into the orifice of the wound in its closed state, as represented in the straight figure; when it reaches the ball, the points D are expanded by pressing the wings near BB with the middle fingers, backwards toward the handle A, lodged in the palm of the hand, thus opening the points, and by a slight motion forwards of the whole Instrument, embracing and securing the substance to be extracted.

## Figures and Tables

**Figure f1:**